# Editorial: Recent Advances in Geomicrobiology of the Ocean Crust

**DOI:** 10.3389/fmicb.2017.01368

**Published:** 2017-07-21

**Authors:** Beth N. Orcutt, Jason B. Sylvan, Cara M. Santelli

**Affiliations:** ^1^Bigelow Laboratory for Ocean Sciences East Boothbay, ME, United States; ^2^Department of Oceanography, Texas A&M University College Station, TX, United States; ^3^Department of Earth Sciences, University of Minnesota Minneapolis, MN, United States

**Keywords:** geomicrobiology, deep biosphere, IODP, ocean crust, iron oxidation

Igneous oceanic crust is one of the largest potential habitats for life on earth (Orcutt et al., 2011), and microbial activity supported by rock-water-microbe reactions in this environment can impact global biogeochemical cycles (Bach and Edwards, 2003). However, our understanding of the microbiology of this system, especially the subsurface “deep biosphere” component of it, has traditionally been limited by sample availability and quality. Over the past decade, several major international programs (such as the Center for Dark Energy Biosphere Investigations, the current International Ocean Discovery Program, and its predecessor Integrated Ocean Drilling Program, and the Deep Carbon Observatory) have focused on advancing our understanding of life in this cryptic, yet globally relevant, biosphere. Additionally, many field and laboratory research programs are examining hydrothermal vent systems—a seafloor expression of seawater that has been thermally and chemically altered in subseafloor crust—and the microbial communities supported by these mineral-rich fluids. The papers in this special issue bring together recent discoveries of microbial presence, diversity, and activity in these dynamic ocean environments.

Starting at the seafloor where igneous rocks are directly exposed to oxic, bottom seawater, two papers in this special issue address the microbial diversity (Lee et al.) and metagenomic characteristics (Singer et al.) of seafloor basalts. Going deeper below the seafloor where cool, oxic fluids circulate through fractures in the lithosphere, two papers document the biomass and structure (Jørgensen and Zhao) and metabolic potential (Zhang et al.) of biofilms on subsurface basalts from the western flank of the Mid-Atlantic Ridge at a site known as North Pond. A companion project at this same site used a new *in situ* spectral imaging tool to assess biofilms and biomass in the crustal subsurface (Salas et al.). Basalts from the seafloor and this oxic subsurface environment were used in a survey to assay the magnitude of microbial carbon fixation on basalts, and to identify microbial groups potentially involved in this process (Orcutt et al.). Isolation and description of bacteria from the overlying sediment at North Pond provides a comparison between the crustal microbial communities and those detected in the sediment lying just above (Russell et al.).

Comparing these oxic and cool subsurface crustal fluids with warmer and anoxic subsurface crustal fluids from the eastern flank of the Juan de Fuca Ridge, a new nanocalorimetry approach documents the energy available to subsurface crustal fluid communities (Robador et al.). These new approaches help to validate theoretical models of energy availability in the crustal subsurface, such as new work in this special issue on hydrogen production from water-rock reactions (Bach) and radiolysis (Dzaugis et al.). Finally, new time series data from a crustal observatory at the Juan de Fuca Ridge flank reveals the importance of redox conditions and temperature on structuring biofilms forming on crustal rocks (Baquiran et al.).

The Juan de Fuca Ridge and flank environment are also loci of recent efforts to cultivate thermophilic organisms. Thermophilic sulfate reducing bacteria were isolated from deep sediment on the ridge flank influenced by the diffusion of sulfate and heat from the underlying igneous basement (Fichtel et al.), and multivariate laboratory experiments were conducted to determine controls on thermophilic sulfate reduction in the hydrothermal sulfide chimney structures nearer to the ridge axis (Frank et al.). The genomes of isolates of *Thermococcus* from the Juan de Fuca were compared to isolates from other hydrothermal systems to explore thermophile biogeography and adaptation (Price et al.).

Moving to a more organic-rich hydrothermal setting, two papers in the special issue explore the microbial residents in the Guaymas Basin in the Gulf of California. A “hiking guide” of Guaymas documents the connection of surface patterns in microbial mats to subsurface gradients in temperature and porewater chemistry (Teske et al.). Honing in on the surface microbial mats, and the resident giant sulfur bacteria therein, an exploration of genomes from these organisms reveals intriguing hints about their possible transcriptional regulation mechanisms (MacGregor).

Given that oceanic crust is composed of igneous rocks (e.g., basalt, gabbro, peridotite) enriched in reduced iron, chemosynthetic iron oxidation received focused attention in this special issue. The growth of chemoorganotrophic (Sudek et al.) and chemolithoautotrophic (Henri et al.) iron-oxidizing bacteria in the presence of basalt was investigated with interdisciplinary approaches. Two studies show the early colonization by marine Zetaproteobacteria—neutrophilic, microaerophilic iron-oxidizing bacteria—on surfaces containing reduced iron (Henri et al.; McBeth and Emerson). Zetaproteobacteria also colonize reduced iron substrates in freshwater systems (McBeth and Emerson), and genomes of freshwater iron oxidizing bacteria reveal similarities and differences to their marine cousins (Kato et al.). An exploration of the architecture of the microbial mats generated by freshwater and marine iron-oxidizing bacteria reveals the ecosystem structuring performed by these lithotrophs (Chan et al.). Finally, an intense investigation of the alteration products in active and inactive hydrothermal chimneys reveals diverse signatures of microbial iron oxidation and reduction (Toner et al.).

Cumulatively, the articles in this special issue serve as a tribute to the late Dr. Katrina J. Edwards (Figure 1), who was a pioneer and profound champion of studying microbes that “rust the crust” (Bach and Edwards, 2003; Edwards et al., 2003, 2005, 2011a,b, 2012a,b). As co-author on five of the twenty-two papers in this special issue (Lee et al.; Salas et al.; Singer et al.; Baquiran et al.; Toner et al.), and an acknowledged inspiration of several others, Katrina's influence on the field has a lasting legacy. This legacy is eloquently captured in an award-winning feature length documentary about the North Pond ocean drilling project (https://vimeo.com/117447690), a long-term observatory project that Katrina initiated to study deep biosphere crustal microbes (Edwards et al., 2012c). Her legacy lives on in the various collaborators that continue to study microbes that rust the crust, as well as in the scientists that passed through her lab and are now running their own labs, including the editors of this volume. This special issue volume serves as a foundation for the continued exploration of the subsurface ocean crust deep biosphere.

**Figure 1:**
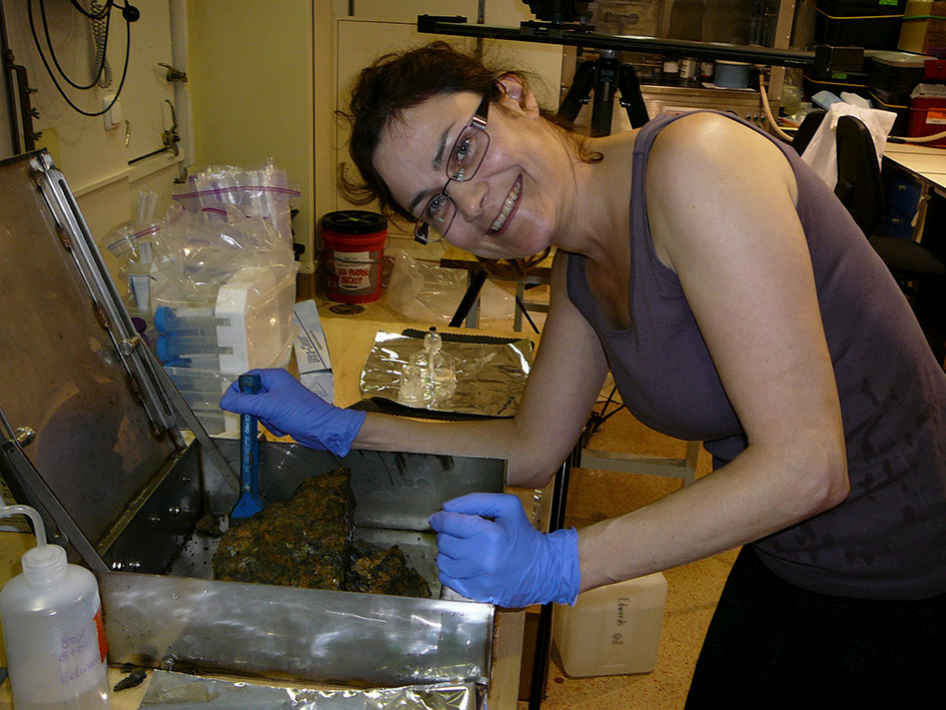
Dr. Katrina J. Edwards (1968–2014), a visionary geomicrobiologist who promoted the study of microbes that “rust the crust,” breaking open a seafloor basalt from the North Pond study site in 2012. Photograph by Beth Orcutt.

## Author contributions

All authors listed have made a substantial, direct and intellectual contribution to the work, and approved it for publication.

### Conflict of interest statement

The authors declare that the research was conducted in the absence of any commercial or financial relationships that could be construed as a potential conflict of interest.
